# Identifying childhood malaria hotspots and risk factors in a Nigerian city using geostatistical modelling approach

**DOI:** 10.1038/s41598-024-55003-x

**Published:** 2024-03-05

**Authors:** Taye Bayode, Alexander Siegmund

**Affiliations:** 1 Institute of Geography & Heidelberg Centre for Environment (HCE), Heidelberg University, Heidelberg, Germany; 2 Department of Geography-Research Group for Earth Observation (rgeo), UNESCO Chair on World Heritage and Biosphere Reserve Observation and Education, Heidelberg University of Education, Heidelberg, Germany

**Keywords:** Urban health, Spatial variability, Childhood malaria, Geostatistics, Exceedance probability, Diseases, Risk factors, Socioeconomic scenarios, Preventive medicine

## Abstract

Malaria ranks high among prevalent and ravaging infectious diseases in sub-Saharan Africa (SSA). The negative impacts, disease burden, and risk are higher among children and pregnant women as part of the most vulnerable groups to malaria in Nigeria. However, the burden of malaria is not even in space and time. This study explores the spatial variability of malaria prevalence among children under five years (U5) in medium-sized rapidly growing city of Akure, Nigeria using model-based geostatistical modeling (MBG) technique to predict U5 malaria burden at a 100 × 100 m grid, while the parameter estimation was done using Monte Carlo maximum likelihood method. The non-spatial logistic regression model shows that U5 malaria prevalence is significantly influenced by the usage of insecticide-treated nets—ITNs, window protection, and water source. Furthermore, the MBG model shows predicted U5 malaria prevalence in Akure is greater than 35% at certain locations while we were able to ascertain places with U5 prevalence > 10% (i.e. hotspots) using exceedance probability modelling which is a vital tool for policy development. The map provides place-based evidence on the spatial variation of U5 malaria in Akure, and direction on where intensified interventions are crucial for the reduction of U5 malaria burden and improvement of urban health in Akure, Nigeria.

## Introduction

Infectious disease like malaria has been a public health burden for generations. Though there have been tremendous advances in its management and treatment, but the public health challenge still lingers. According to the recent World Malaria Report^1^ progress towards fighting malaria is being stalled as there was an increase in malaria cases for the second consecutive year. However, some improvement of 1% fewer malaria-related deaths were recorded in 2021. In 2018, Sub-Saharan Africa (SSA) accounted for 94% of global malaria deaths. Furthermore, children under the age of five (U5) accounted for 70% of malaria-related mortality in the SSA region^2,3^. An increase to 96% of malaria-related death is recorded in WHO African Region in 2021, and the top 16 malaria-affected countries are all situated in SSA. While pregnant women are at heightened malaria exposure risk, about 80% of malaria deaths were from U5 in WHO African Region^1^.

From these worrisome malaria burden statistics, Nigeria takes a large share of the global numbers. In 2021, Nigeria accounted for about 26.6% of malaria cases and 31.3% of malaria-related deaths globally^1^. Descriptively, this amounts to over 50 million and 100,000 of malaria cases and deaths respectively in Nigeria. As averred by^4,5^, about 60% of outpatient hospital visits can be attributed to malaria in Nigeria. In Nigeria, U5 children are the most vulnerable group—they experience about an average of 2–4 bouts per year, and account for about 90% of national mortality from malaria^6^. Furthermore, Nigeria accounted for 38.4% of global malaria deaths in children under five^1^. In case of severe type of malaria, comorbidity such as anaemia, respiratory distress and prostration can be experience by the child^6^.

Coupled with the recent slow progress in malaria reduction in SSA, the recent global pandemic—coronavirus disease (COVID-19)—has further contributed to the interruption of malaria control undertakings in malaria endemic regions of the world. Park et al.^7^ reported the high levels of surfeit malaria morbidity and mortality in Low and Middle Income Countries (LMICs), which could be attributed to poor community engagement and limited malaria tests. For example, the work of Ilesanmi, Afolabi and Iyiola^8^ identifies limited acquisition of malaria tests to healthcare providers as a barrier against visiting health facilities. This could have been because of less funding going towards malaria control because of COVID-19^8^. Thus, the pandemic worsened the healthcare problems such as already weak health systems, ineffective and inefficient health management, and inequitable distribution of human resources between urban and rural areas in Nigeria identified by^9^.

This study sets out to estimate the burden of U5 malaria and variability in the rapidly urbanising medium-sized city of Akure. Overcrowding, environmental degradation, and likely substantial increase in malaria transmission are challenges of rapidly urbanising areas or places in Nigeria such as Akure^10,11^. In Nigeria, small or fine scale (e.g. cities) level variations in the burden of malaria and malaria risk factors are not yet sufficiently understood. National or regional-level surveys may not capture intra-urban specific characteristics and risks of malaria burden^10^. Furthermore, national or regional surveys may miss out on adequate sample sizes or tilt to those who use public health facilities and largely exclude socioeconomic data, behaviours, and a well-defined catchment population^12^. Some studies have explored the risk factors of malaria in Nigeria, however, mostly with the use of descriptive and regression statistical techniques to assess a combination of data from blood testing and questionnaires^13,14^. A few studies have attempted spatial risk analyses of malaria in Nigeria. For example, using Kriging to develop predictive risk factor maps,^15^ assessed the spatial distribution and socio-demographic risk factors of U5 malaria in Nigeria. A close attempt at spatial statistical modelling of malaria incidence and hotspots was made by^16^. These authors used Moran’s diagram, index of local Moran’s I, and spatial regression models to conduct a spatiotemporal analysis of the association between malaria incidence and environmental predictors in Nigeria. In particular^17^, applied Bayesian geostatistical technique to model malaria risk in Nigeria using malaria indicator survey (MIS) data and environmental/climatic data. These studies have all been done at national level which could mask small/local scale spatial variation. Hence, there is sparse use of spatial predictive modelling and the development of probability models with certainty levels to guide the deployment of limited public health resources at sub-national scales in Nigeria which is one of the vital applications of Model-Based Geostatistics (MBG). MBG is a known risk mapping approach which provides robust information on the spatial distribution of infections and facilitates the design and implementation of intervention or control programmes^18^. In addition, MBG modelling method have the capacity to deliver expected precision result for improved decision-making^19^. According to literatures, Model-Based Geostatistics (MBG) is considered as well-established statistical tool for modelling spatial correlation generated by unmeasured risk factors to predict disease prevalence in location of interest or investigation^20,21^. MBG is a principled likelihood-based approach with effective applicability in low resource settings and places characterised with incomplete or non-existent disease registries. With MBG, it is possible to provide probability metrics or quantification for pragmatic policy relevant thresholds. Furthermore, MBG allows for quantification of uncertainty and intrinsic variability in small area predictions^22^. Hence, our assessment is sacrosanct and provides city-level information that contributes to understanding specific characteristics of the area (place) and the people (residents) of such places. Till date and to the authors knowledge, no known works have used MBG explicitly to model the fine scale spatial variation of malaria risk and estimation particularly in Nigeria. Our study aims to fill this gap with the aim of identifying U5 malaria prevalence hotspots while considering the social determinants of malaria which are often not available because of incomplete or lack of health registry especially when local scale is concerned. Our study is significant in supporting public health planning by unveiling areas of high malaria prevalence and associated risk factors. This will lead to allocating already scarce resources necessary to reduce malaria's burden in malaria hotspots.

We recognise that spatial dimensions are crucial when managing infectious diseases. Also, as countries are experiencing a reduction in malaria burden, spatial targeting of the disease control efforts towards malaria risk factors and high-risk locations, which our study supports, is pertinent. Identifying hotspots based on the level of certainty and uncertainty, which the MBG affords us, increases our findings’ usefulness for further research, health policy formulation, decision-making, resource planning, allocation, and implementation. Specific gains include the distribution of limited health resources in particular places where they are most required. We expect that our study will create the needed awareness of using MBG in disease modelling for resource-scarce regions to identify disease hotspots and probability levels for increased attention.

## Methods

### Study setting

**Figure 1:**
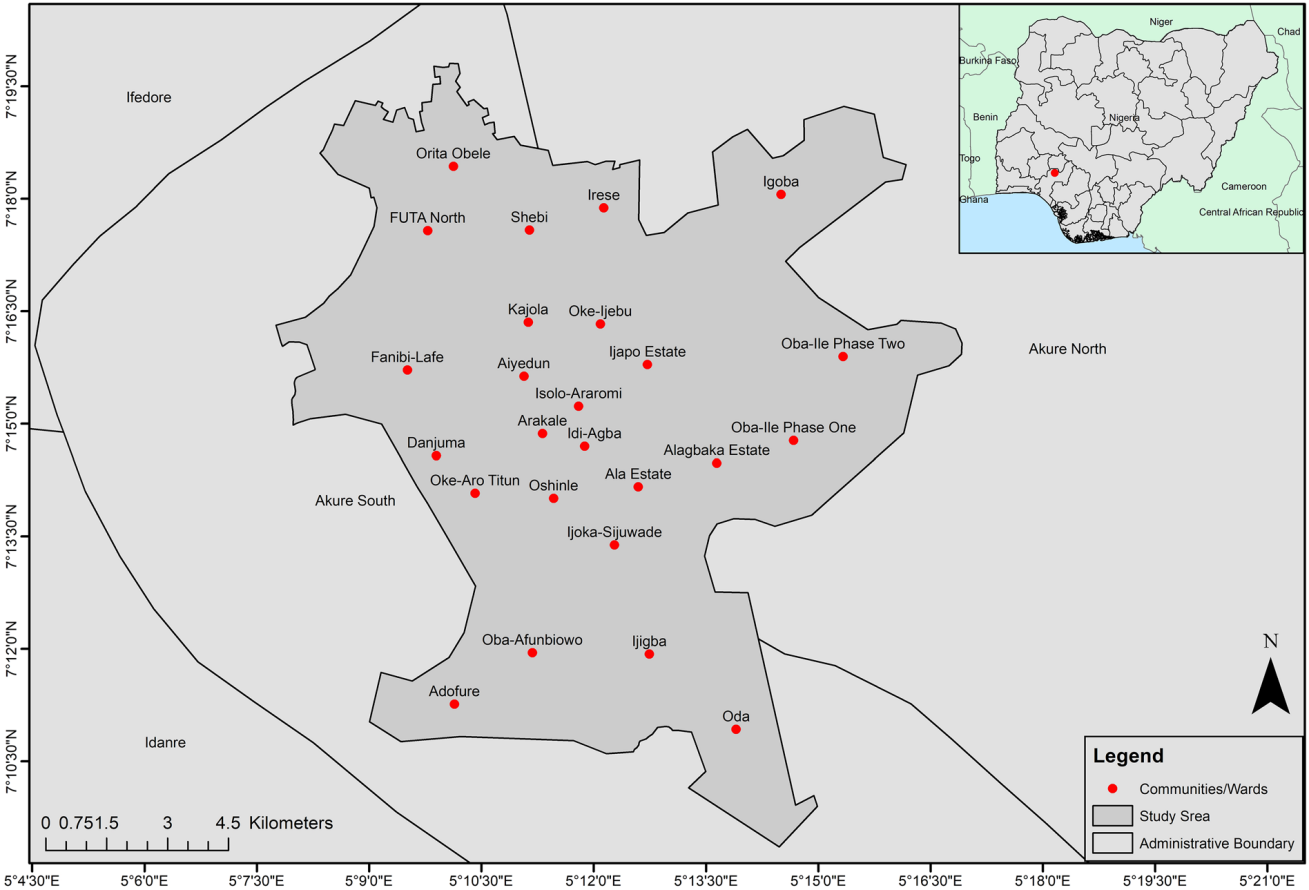
The city of Akure and communities in national context. (Note: The map was drawn by the author with ArcGIS 10.4.1, Esri Inc, http://ww.esri.com. The Nigeria administrative boundaries were gotten from https://datacatalog.worldbank.org/search/dataset/0039368; the boundaries for other countries were gotten from https://datacatalog.worldbank.org/search/dataset/0038272/World-Bank-Official-Boundaries).

The study area, Akure is a medium-sized rapidly urbanising city of Ondo State, which is one of the south-western states of Nigeria as shown in Fig. 1. The fusion of two Local Government Areas (LGA)—Akure North and Akure South—makes up Akure. Since the city became the capital of Ondo State in 1976, several other factors such as being the seat of government, home to the Federal-government owned tertiary institutions such as University of Technology and a College of Agriculture, well-connected transportation routes with proximity to Idanre Hills (a famous tourist centre), have collectively attributed to making Akure the most populated and developed city in Ondo State.

According to (*Population and Housing Census 2006*, n.d.), the population of Akure increased from 239,124 in 1991 to 353,211 in 2006^23^. Since the 2006 census is not reliable^24^, we adopted a practical and reliable estimation from the place-based Geographic, Population and Demographic Data project (https://geopode.world). Based on derived estimate, the city has over one million residents (1,283,541). From the estimate, U5 comprises of about 12% (162,975) of the estimated population. Akure like Ondo State lies in the tropics which is characterised with humid and derived savanna agroecological zones; dry and wet seasons climate^25^ making it a perfect condition for the propagation of vectors (mosquitoes) and transmission of malaria.

### Epidemiologic data and explanatory variables

Epidemiologic data (U5 malaria) for this study was obtained with the aid of a Malaria Indicator Questionnaire (MIQ). U5 malaria was determined by a verbal report based on obtained microscopy/clinical test from health centres/laboratories and response to malaria prescribed treatment. We strictly adopted combinations of these two criteria to reduce our bias about the definition of malaria since we do not have the ethical right, qualification, and skills to carry out malaria test on our study participants. To further reduce bias in our studies, cross-checked questions were included. The purpose of some of these questions is to limit the chances of false confirmation of diseases with similar malaria symptoms according to the studies of^26,27^. Furthermore, the MIQ was utilised to capture malaria explanatory variables within the frame of social determinants of health (SDH) similar to the study of^28^. The considered SDH variables are within the scope of socio-demographic characteristics (child sex, child age, ethnicity etc.); socioeconomic characteristics (household income, father’s education, mother’s education etc.); preventive behaviour (insecticide-treated bednets—ITN, availability of health infrastructure etc.); built-environmental factors (Window protection, covered roof eaves etc.); and the environmental health factors (drainage condition and covering, toilet facility, proximity to waste disposal point etc.). The considered variables for the analysis were determined after considering the extensive works of^28–30^. MIQ is known to be effective in places of low disease reporting rate and paucity of malaria data^29,31^.

### Sample size and sampling technique

In most cases, available secondary malaria data from hospital visits lack the important characteristics (socio-economic status (SES) and sociodemographic) and absolute spatial reference (coordinates) thereby making such data unsuitable for our study objectives. These peculiarities are prevalent in SSA particularly in local settings/scale and Akure is no exception. To deal with this challenge, we randomly sampled 1000 buildings like in the study of^32^, with the hope that we would be able to obtain about 600 valid study participants. The estimation of Nigeria’s population, particularly children below the age of five, poses challenges due to infrequent and biased government censuses^24^. Additionally, identifying households with young children in the country beforehand is nearly unfeasible due to lack of antecedent knowledge of houses or households with U5 children. To address these constraints, our study leveraged previous research to determine the sample size, taking budgetary limitations into account. We utilized building sampling as a spatial reference to locate households during our field visits. By importing the extracted building data into ArcGIS Pro, we were able to generate accurate locations of the sampled buildings in relation to the GPS coordinates used during our field survey. The buildings in Akure were extracted following the methodology described on the Picterra platform with a paid subscription (https://picterra.ch/geospatial-imagery-analysis).

The study samples are within the scope of other cross-sectional studies and population proportion sampling method of^33,34^. According to our knowledge of local demography, most households with U5 children have only one child under five years. In rare cases where there were more than one U5 child i.e. two, we selected the youngest and subsequently selected the eldest in next household with such a similar characteristic as our aim is to model individual-level variability of childhood malaria in Akure. With random sampling, each child, house, or household has equal chances of being selected thereby reducing the risk of selection bias.

### Data collection and informed consent

The lead author assisted with five research assistants visited each of the pre-identified houses with the MIQ to gather evidence on active malaria cases after the rainy season. The survey period for this study was between October and December in the year 2019. According to^35^, dry season in Akure is from November to March while the rainy season is from April to October. The sampled houses (families) were visited between 4:30 pm and 6:00 pm to enhance effective targeting of the respondents. Upon visiting a sampled location, the guardian, parents, or adult relative (> 18 years) with the child’s health history was interviewed. Privacy and ethical consent procedures were observed and strictly followed. We obtained informed consent from guardians, parents or adult relative to participate in the study or partake in the interview. Furthermore, we assured, maintained, and adhered to the anonymity of the data and presentation of results obtained from analyses of collected data. The MIQ for the study was created in English language, however, with the option of conducting the interview in Yoruba language (native language in Akure) in case a guardian/parent has a low level of English literacy. This approach ultimately improved the level of inclusion in this study since the lead author and field assistants understands English and Yoruba language. This research was performed in accordance with relevant guidelines and regulations. The methods of data collection and interpretation are in accordance to declaration of Helsinki ethical principles and codes.

### Exploratory analysis

Before the development of the geostatistical models for this study, we preliminarily carried out an exploratory analysis of the data. The purpose of this is to provide insights and guides into development of best fit geostatistical model for U5 malaria prevalence^36^. The objectives and focus on this stage of analysis are:

(i) Establish the determinants variables or factors of U5 malaria prevalence. This can be accomplished by utilisation of bivariate analysis such as Chi-square (${\mathcal{X}}^{2} )$ to build a table summary of the association between U5 malaria and the covariates. This formed the basis of non-spatial analysis discussed in the later section of this paper.
(ii) Explore the association between U5 malaria prevalence and covariates i.e. explanatory or independent variables. In this stage, we fitted a non-spatial generalised linear model (GLM) to observed and assess the relationship (magnitude and direction) of the covariates with U5 malaria prevalence. The selected model has the least Akaike Information Criterion (AIC) from the stepwise forward approach that was conducted. In addition, Variance Inflation Factor (VIF)/generalized Variance Inflation Factor (GVIF) was used as regression diagnostic measure to detect the presence of collinear variables in order to avoid multicollinearity in our model and reduce standard error of model coefficients according to the works of^37,38^. Furthermore, we evaluated our designed model accuracy using cross-validation (*k*-fold) technique. The purpose of this is to test the effectiveness of our model against data points which were not used during the training of the model (new data sets). During the model training randomly selected subset of the data (training set) is used to inform predictions at location of remainder of the data (test set)^36^. The combination of these methods (GVIF and cross-validation) guide against correlation among model explanatory variables, overfitting of our model, evaluate prediction accuracy, and provide insight on variable importance and selection asides the retention of variables based on their p-values (p < 0.05). In the final step, the odds ratio (OR) which determines risk factors of U5 was computed. Given the exposure or factor, OR greater than 1 means the U5 malaria is likely to occur; OR less than 1 means the event (U5 malaria) is less likely to occur while OR equals 1 means the likelihood of malaria does not change.
(iii) Examine spatial dependency of U5 malaria by testing for spatial correlation on the residuals i.e. to examine spatial dependency in step (ii). The focus is to determine if variation in the residuals i.e. variation that is not captured by the retained variables reveal evidence of spatial correlation by using empirical variogram^36^. The choice of spatial model is determined by the detection of spatial correlation in the residual*.*

### Geostatistical modelling

Unlike non-spatial/standard statistical modelling, spatial data and modelling observe the assumption of spatial dependence (autocorrelation) between neighbouring locations due to observed common exposure^39–41^. Spatial autocorrelation in this context refers to the relationship between U5 malaria of a child (*Y*_*ij*_) in location *j* with itself in another neighbouring location within the same geographical space^39^. Spatial autocorrelation expresses the degree of similarity among the observation values within the geographical space of interest^42^. Therefore, to account for spatial dependency, we formulated a geostatistical model which follows the geostatistical model for prevalence surveys by^43^. The model is within the generalised linear mixed model framework or spatial generalised linear mixed models (SGLMMs) which relates disease prevalence data with potential linear predictors, binomial error distribution, logistic-link function and latent Gaussian process by adding random effects at the observed locations^43,44^. Model-based geostatistics has its origin from Kriging which is a method of interpolating (predicting values at unmeasured locations) or smoothing spatial data. Particularly, MBG is termed as application of explicit parametric stochastic models and likelihood-based methods of inference to geostatistical problems^45^. The interpolation is based on observation data pairs while correlation is a function of distance between the data pairs^43,46^.

Equation (1) describes the likelihood-based Binomial Geostatistical Model adopted for this study. This is an extension of a binary logistic regression model by the inclusion of random effects and spatially correlated random effects i.e. spatial Gaussian process. Hence, let U5 malaria status Y_ij_ of a child* i* at location *j* take the value of 1 if a child has malaria, and 0 otherwise. The dependent variable—Yij follows a Bernoulli probability distribution with P(Y_ij_ = 1) = ${\mathcal{P}}_{ij}$ which is conditional on a stationary Gaussian process $\left( x \right){ }$ and an additional set of study location specific and unobserved random effects $Z_{i}$, the linear predictor of the model assumes the form:

1 $$\log \left( {\frac{{P_{ij} }}{{1 - P_{ij} }}} \right) = d\left( {{\mathcal{X}}_{i} } \right)^{\prime } \beta + \left( {{\mathcal{X}}_{i} } \right) + Z_{i}$$

where $${\mathcal{X}}_{i}$$ is the vector of a child, with individual-level covariates with associated regression coefficient $$\beta$$; S = {$$\left( x \right)$$: $$x$$ $$\in$$ R^2^} is a Gaussian process with mean zero, variance $$\sigma^{2}$$, and correlation function *p*($$x,x^{\prime}$$) = Corr {$$\left( x \right)$$,S($$x^{\prime}$$)}. The Gaussian process ($$S)$$ is stationary and isotropic, while the correlation function is a function of euclidean distance^47^. The aim of study location random effects $$Z_{i}$$ is to account for the unexplained nonspatial variation which could be small scale spatial variation or measurement error. This is also known as the nugget effect ($${\uptau }^{2} )$$. The random effects are independent normal, (i.e. Zi ~ N (0, $${\uptau }^{2}$$)) variates.

In Eq. (1), we write $\tau^{2}$ for the variance of $Z_{i}$ and model S $\left( x \right)$ as a stationary Gaussian process with variance $\sigma^{2}$ and matérn correlation function^48^. Matérn model is an efficient method for modelling correlation function as strongly recommended by^45,49,50^. It contains kappa (k) which determines the smoothness of the process. The matérn correlation function is given by:

2 $$\rho \left( {u;\emptyset ,\kappa } \right) = \frac{{2^{\kappa - 1} {\Gamma }_{\left( \kappa \right)} }}{{\left( {u{|}\emptyset } \right)^{\kappa } \kappa_{\kappa } \left( {u{|}\emptyset } \right)}},\;\;u > 0,$$

where $\emptyset$ > 0 is a scale parameter which regulates the rate at which the spatial correlation goes to zero or decays as the distance increases^51,52^; k > 0 is the shape parameter which determines the smoothness of $\left( x \right)$. Kk (.) is the modified Bessel function of the second kind of order k > 0, and $u$ is the distance between two sampled locations. Kappa is difficult to estimate reliably since this will involve large data collected at small distances. Hence three discrete set of values (0.5, 1.5, 2.5) corresponding to different level of smoothness have been defined for Kappa^44^. These values correspond to the discontinuity of the different level of smoothness. For this study, we adopted 0.5 for Kappa according to the documentation and works of^36,44^. Kappa of 0.5 corresponds to exponential correlation function i.e. the Matérn covariogram becomes the exponential one^44,53^. Furthermore, most functions available in PrevMap package in R, the Matérn shape parameter $\kappa$ is treated as fixed because not all parameters in the Matérn class can be estimated consistently. Matérn class has the capacity to model the behaviours of variogram and it consists of exponential variograms as a special case unlike other popular covariograms such as exponential, powered-exponential, gaussian or spherical covariograms. For more technical details, we refer the reader to the works of^44,46,53^.

### Monte Carlo maximum likelihood and spatial prediction

In this study, Monte Carlo maximum likelihood methods (MCML) was utilised for parameter estimation as documented in the PrevMap package in R^44^. MCML is based on importance sampling techniques approximation of the high-dimensional intractable integral that defines the likelihood function^54^. It enables flexibility in fitting complex models and avoids asymptotic inference and computational challenges encountered in solely likelihood-based fitting^55^. The likelihood function for parameters $\beta$ and $\theta^{{\text{T}}}$ = ($\sigma^{2}$, $\emptyset , {\uptau }^{2}$) is obtained by integrating out the random effects included in Eq. (1), where $\sigma^{2}$ is the variance, $\emptyset$ is the range, and ${\uptau }^{2}$ is the nugget effect. We map the risk of U5 malaria over 100 × 100 m grid. Spatial distribution maps of U5 malaria prevalence, likelihood-based geostatistical modelling and spatial prediction were developed in R statistical programming (R version 3.6.3). To improve the model predictions, the covariates are included. The selected covariates for the spatial model were carefully considered according to their significance level as discussed in earlier section i.e. Exploratory analysis. Often, the development of public health policies are based on the exceedance, or non-exceedance of a predefined prevalence or incidence thresholds say *t*^36^. Therefore, the exceedance probability (EP) of U5 malaria prevalence predictions in each location above the predefined thresholds *t* can be expressed or defined as:

2 $${\text{EP}}({\mathcal{X}}) = {\text{Probability}}\left[ {{\mathcal{P}}\left( {\mathcal{X}} \right){ > }\left. {\text{t}} \right|{\text{Y}}_{1} , \ldots ,{ }\left. {{\text{Y}}_{{\text{N}}} } \right]} \right..$$

It is necessary to note that the resulting estimates at each locations have uncertainties that need to be taken to consideration^52^. The exceedance probability can help to overcome this challenge and prevent unjustifiable policy decisions by quantifying how likely ${\mathcal{P}}\left( {\mathcal{X}} \right){ }$ is to be above a threshold *t* as shown in Eq. (3). For this study, we set prevalence threshold to be 10% (0.1) which can be categorised as hotspots of U5 malaria in Akure. According to^56^, places with annual malaria prevalence of 10–35% have moderate transmission while area of high transmission are above 35%. However, the recently implemented fifth National Malaria Strategic Plan (NMSP) covering the period of 2021–2025 in Nigeria aims to achieve parasite prevalence of less than 10%^57^. We therefore adopted 10% as our exceedance threshold to determine hotspots of malaria prevalence in Akure. If EP is close to 100%, this shows that U5 malaria prevalence to be above the threshold *t* is very high*;* if EP is close to 0%, the prevalence of U5 malaria is highly likely to be below *t.* EP close to 50% suggests high level of uncertainty which means that prevalence of U5 malaria is equally likely to be above or below *t.*

### Ethical approval

We received ethical approval from the institutional review board at the Institute of Geography, Heidelberg University, Germany. We obtained informed consent from parents, guardians or adult relative who participated in the interview, and we adhered to the anonymity of the data and presented results. Before the interview was carried out, ethical clearance was obtained from the Ondo State Ministry of Health.

## Results

### Non- spatial analysis

We effectively obtained about 60% valid responses (n = 568). As mentioned earlier, we do not have previous knowledge of houses or households with U5 children. This has contributed to the low responses coupled with budget constraints to sample more houses. Furthermore, we expunged participants who had spent less than two weeks in the location depending on the week of survey to reduce risk of imported malaria. Nevertheless, the obtained valid responses were deemed sufficient after carrying out statistical power analysis with open source G*Power tool, version 3.1.9.6^58^. The point prevalence of U5 malaria in Akure, Nigeria based on verbal confirmation according to the study definition of positive malaria was 22.5%. Malaria prevalence among the female children (23.3%) is higher compared to malaria prevalence among male children (21.9%) according to Table 1. According to the study, about 40% of the children have ITN. Further to the study findings in Table 1 on the usage of ITN and its impact on malaria prevalence, children who sleep under ITN have lower prevalence of malaria (16.7%) compared to children who do not sleep under ITN (26.4%). This further implies that usage of ITN is malaria risk factor with significant reduced odds of U5 malaria and serves as protection against mosquito bites.

**Table 1:** Description of U5 malaria with considered covariates.

Factors	Negative (N = 440)	Positive (N = 128)	OR (95% CI)	p value
Child sex				0.708
Female	198 (76.7%)	60 (23.3%)	1.00	
Male	242 (78.1%)	68 (21.9%)	0.93 (0.62–1.37)	
ITN				0.007
No	251 (73.6%)	90 (26.4%)	1.00	
Yes	189 (83.3%)	38 (16.7%)	0.56 (0.36–0.86)	
Urban agriculture				0.351
No	321 (78.5%)	88 (21.5%)	1.00	
Yes	119 (74.8%)	40 (25.2%)	1.23 (0.79–1.88)	
Waste disposal				0.457
Burning	128 (75.3%)	42 (24.7%)	1.00	
Water body	4 (66.7%)	2 (33.3%)	1.52 (0.27–8.61)	
Others	1 (50%)	1 (50%)	3.05 (0.19–49.8)	
Pick up	231 (77.3%)	68 (22.7%)	0.90 (0.58–1.39)	
Vacant plot	76 (83.5%)	15 (16.5%)	0.60 (0.31–1.16)	
Child age				0.352
< 1 year	38 (88.4%)	5 (11.6%)	1.00	
1–2 years	79 (77.5%)	23 (22.5%)	2.21 (0.78–6.27)	
2–3 years	94 (78.3%)	26 (21.6%)	2.10 (0.75–5.88)	
3–4 years	98 (73.1%)	36 (26.9%)	2.79 (1.02–7.64)	
4–5 years	131 (77.5%)	38 (22.5%)	2.20 (0.81–5.99)	
Window protection				0.001
No	125 (69.1%)	56 (39.9%)	1.00	
Yes	315 (81.4%)	72 (18.6%)	0.51 (0.34–0.77)	
Water source				0.007
Dug well	198 (72%)	77 (28%)	1.00	
Other	68 (79.1%)	18 (20.9%)	0.68 (0.38–1.22)	
Piped water	174 (84.1%)	33 (15.9%)	0.48 (0.31–0.77)	

The study findings show that vector-proof houses are determinant factor of malaria. Vector-proof houses protect against malaria. The houses in good condition characterised with good window screening have a lower prevalence of malaria (18.6%), while children living in substandard houses characterised with poor or defected window covering recorded higher prevalence of malaria (39.9%). The condition and source of drinking water also plays significant role in the burden of malaria. According to the study findings, almost half (48%) of the survey households depend on Dug well as water source. Despite this large figure, the burden of U5 malaria is higher (28%) among households with Dug wells compared to affluent households that depends on piped water source (15.9%). As shown in Table S1 (supplementary file), drainage with covering is a determinant and risk factor of malaria. U5 children living in places with covered drainage records less burden of malaria (13.3%) compared with U5 children dwelling in places with poor drainage facilities (24.3%). According to Table S1 (supplementary file), Education, Income and type of employment further illustrate effect of social determinants or socioeconomic characteristics on health. U5 children whose fathers are employed in the formal sector have lower burden of malaria (18.2%) compared with U5 children whose fathers are either work in informal sector (26%) or unemployed (28.6%). This phenomenon is similar to the study findings on effect of income level on U5 malaria. According to our study findings, the burden of malaria reduces as income level increases (Table S1 as supplementary file). U5 malaria prevalence is lower among mothers who have obtained tertiary education (18.4%) compared to mothers with no education (33.3%).

Supplementary Table S1 online contains additional table summary of mostly non-significant covariates in this study. We have discussed some selected covariates in the manuscript.

### Model results

The results reported in Table 2 describes the significant predictors and parameter estimates from the binomial logistic model for this study as documented in Eq. (1). The sigma sq $(\sigma^{2} )$ is the variance of the Gaussian process,  $\emptyset$ is the scale parameter which represents the extent of the spatial correlation in metres, while tau sq (${\uptau }^{2} )$ is the non-spatial variation. After further exploration of the model results particularly because of the binary response at each sampled locations we fitted the model without Z terms i.e. tau sq (${\uptau }^{2} )$. This pragmatic decision further led to the improvement of the model fit. According to the model result, the model accuracy from the *k*-fold cross-validation was 0.75 (75%) and Cohen’s kappa was 0.01, which could be considered “slight” given the thresholds of^59^ relatively indicating good performance. ITN, window protection and piped water source are significant with high variable importance. In addition, these variables are not correlated to each other according to the GVIF values (see Table 2). Therefore no added uncertainty in the model estimates and almost non-multicollinearity have been maintained since the VIF values are very close to 1 and lower than threshold of 5 as explicitly discussed in^60,61^. The usage of ITN reduces the risk of malaria burden. Concurrently, vector-proof houses with good window protection have a negative relationship with the likelihood of positive malaria outcomes. Water sources (i.e. piped) have a negative association with the probability of malaria, while other sources of water are non-significant.

**Table 2:** Monte Carlo maximum likelihood estimates for the binomial logistic model.

Factors	Estimate	StdErr	p value	GVIF
ITN	− 0.5159	0.1004	2.779e−07***	1.005669
Window protection	− 0.9137	0.1116	2.586e−16***	1.064548
Piped water source	− 0.3985	0.1074	0.0002063***	1.068029
Spatial covariance parameters				
Log $\sigma^{2}$	− 0.2242	0.0696		
Log $\emptyset$	6.4724	0.2191		

***p < 0.001; **p < 0.01; *p < 0.5.

The units of the scale parameter ∅ is in metres.

**Figure 2:**
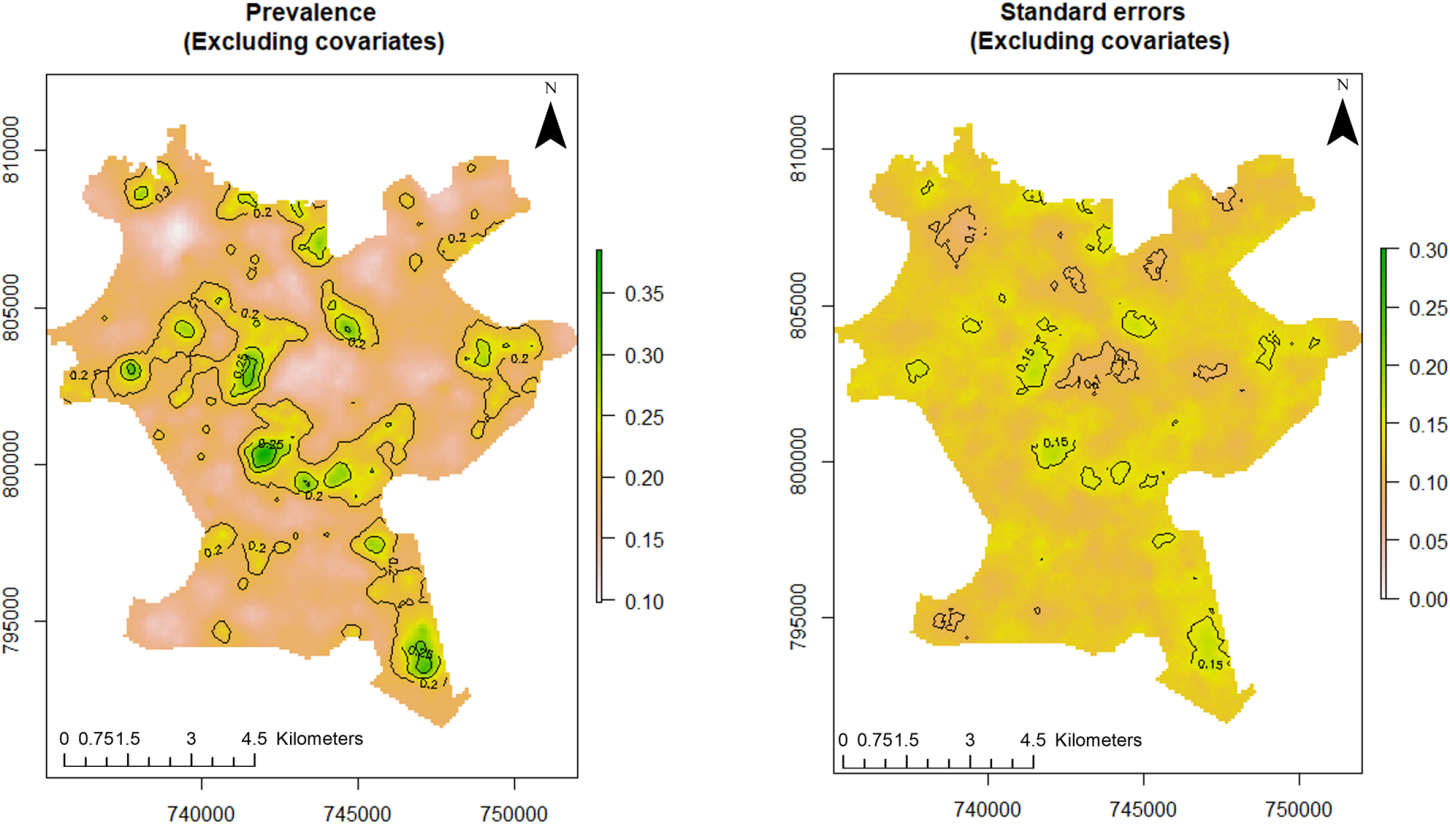
Predictive distribution of U5 malaria in Akure (left panel) and standard errors of the predictions (right panel). The figure was created with R version 3.6.3, https://cran.rstudio.com/.

**Figure 3:**
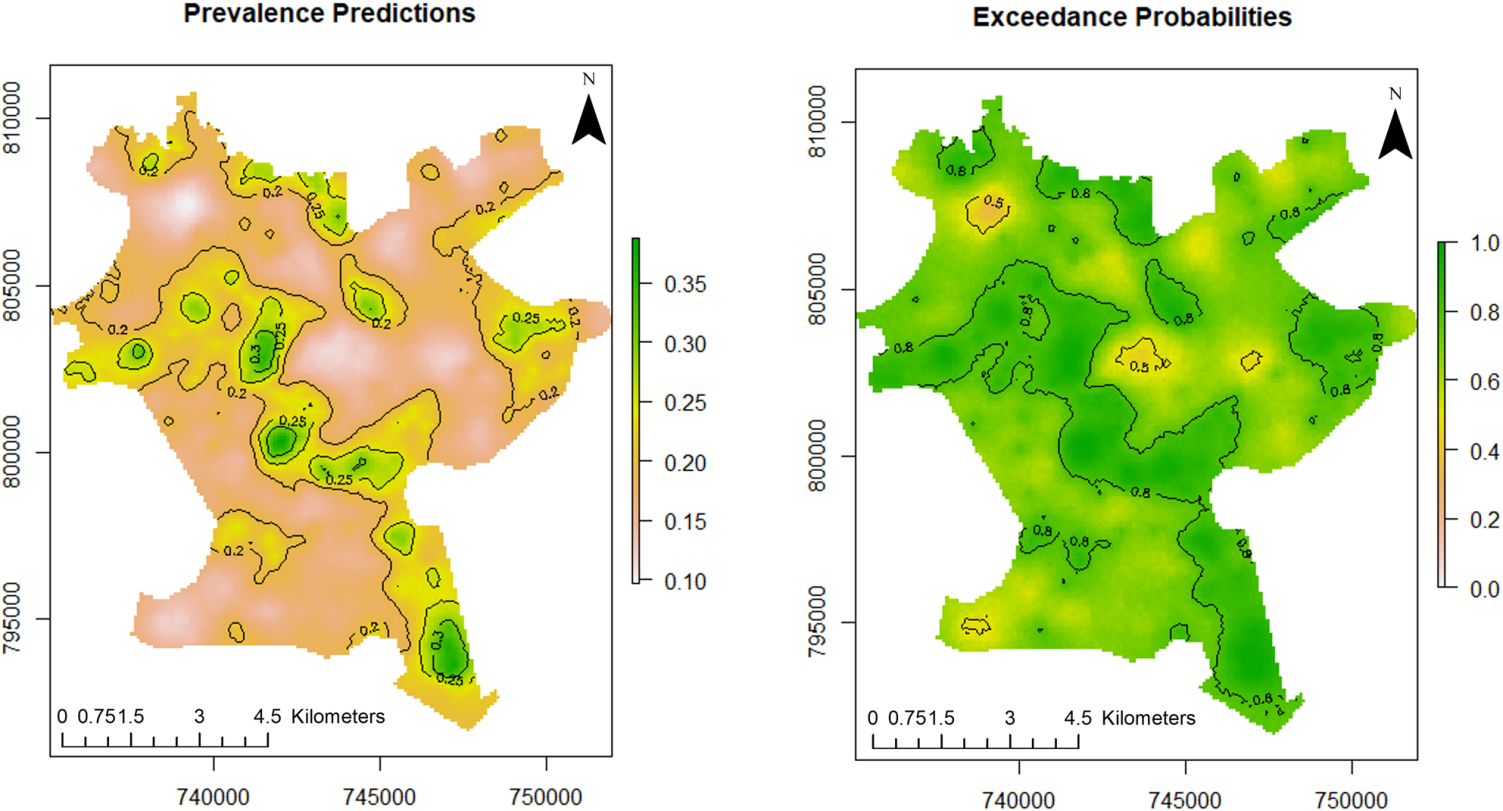
Predictive distribution of U5 malaria in Akure (left panel) and exceedance probabilities (right panel). The figure was created with R version 3.6.3, https://cran.rstudio.com/.

For this study, point referenced U5 malaria prevalence data were analysed using MBG models to outline and map areas where prevalence of U5 malaria is above or below a set policy threshold. We predicted the prevalence of U5 malaria at a fine scale (100 × 100 m resolution map). The predictive power of the model increases when disease predictors are considered. According to Fig. 3 (left panel), the predicted prevalence of U5 malaria in Akure is slightly above 35%, while it is about 35% when the predictors are not considered as seen in the left panel of Fig. 2. Furthermore, the probability that U5 malaria prevalence is above 10% is shown in the right panel of Fig. 3. We used the 10% exceedance threshold to determine hotspots for this study associated with the level of certainty. Therefore, areas with $\ge$ 80% probability of exceeding the threshold were considered hotspots. The certainty level is captured with the contour lines. The uncertainty in the estimates is quantified using the standard errors as shown in right panel of Fig. 2. A set of diagnostic plots that provide checks on the convergence of the MCMC is provided in Fig. S1 (supplementary file).

## Discussion

Spatially targeted policy and healthcare intervention are pertinent to eradicating disease transmission. e.g., malaria. Therefore, spatial modelling of disease remains an important public health tool. Through disease models, hotspots can be determined for prioritising timely intervention in resources-scarce contexts.

The reduction in malaria burden has stalled. The recent figures of global malaria burden according to^1^ is the same level as before 2011, with much increase in the last two years. Furthermore, the national-level statistics may not reflect what is obtainable at lower administrative levels. Since countries are experiencing reduction in malaria burden between 2010 and 2019 as reported by^1^, coupled with the scarce availability of health resources, spatial targeting of intervention for maximum utilisation of resources is essential. Geostatistical methods as seen in this study provides the opportunity for precision in hotspot determination.

There is variability in U5 malaria spatial distribution in Akure. The spatial predicted burden of U5 malaria is higher in the poor and low-income communities such as Arakale, Isolo-Araromi, Ayedun, and Oda. The high malaria transmission might have been due to the lack of suitable housing infrastructure. Based on the morphology of Akure as documented by^62^, Arakale and Isolo-Araromi are communities in the city centre characterised by old and substandard buildings, poor drainage facilities and below-minimum space between buildings. Collectively, these features aid high transmission of malaria. Conversely, Oba-Ile Phase Two and Oda which are newly emerging areas (suburbs) and outlying districts of Akure are also characterised with high burden of malaria as shown in the exceedance probability model (right panel of Fig. 3). These places have prevalence greater than 10% with 80% certainty. These newly emerging places show element of poor planning control^11,24^ with fragmented sites which are suitable vector breeding sites^63^. Lower transmission of U5 malaria was observed in affluent neighbourhoods such as Oba-Ile Phase One, Ijapo Estate and Alagbaka Estate. These areas have standard building structures and better facilities such as good rood conditions, drainage, a good water supply and less vegetation^62^ and robust urban planning development control.

Local spatial estimations of disease allow us to identify locations of disease clusters where disease prevalence is above the geographical average (hotspots). In this study, U5 malaria hotspots were determined through the exceedance probability model as shown in the right panel of Fig. 3. 10% cut off was adopted as the threshold to identify malaria hotspots which is in accordance with the NMSP target set by the Nigerian government. According to the exceedance probabilities model, the dark green areas show locations where U5 malaria prevalence is above 10% with certainty level of 80% and above. These places such as Isolo-Araromi, Arakale, Aiyedun, Kajola, Idi-Agba, Fanibi-Lafe, Oba-Ile Phase Two, Oda, Orita Obele and Irese. The identified places require targeted malaria control effort by the health authorities towards malaria elimination in order to meet the NMSP target.

The study analyses elucidate the risk factors of U5 malaria prevalence. Based on our model results, several factors determine the risk of malaria among U5 in Akure. Although not significant, child’s gender is one of these factors. Male children exhibited a slightly lower malaria burden than their female counterparts. A similar study conducted in Cameron shows a non-significant association between child sex and malaria with a lower burden among male children^64^, while the studies of^65,66^ show significant lower burden of malaria among males compared to females. However, among older children, males are more prone to malaria because of their higher engaging outdoor activities compared to female^13,67^. The reason for our findings could be difference in background immunity between male and female children.

The availability and usage of ITNs is another significant and important factor that affects malaria exposure. According to our study, the usage of ITNs reduces the likelihood of childhood malaria by 56% (OR = 0.56; 95% CI = 0.36–0.86) in Akure, Nigeria compared the children who do not sleep under ITN. Our findings agrees with the following studies in Ghana^30^, Nigeria^68^, Uganda^69^, and Kenya^70^. Good ITN protects against mosquito vectors by reducing the vector-to-human contacts. This mechanically prevents or stops mosquito bites.

The impact of urban agriculture on the susceptibility of malaria among children under five was not significant which is in agreement to the studies of in Ibadan Nigeria^13^ and^71^ in Malawi. According to our study, households that practice urban agriculture are 1.23 times likely to have malaria (OR = 1.23; 95% CI = 0.79–1.88) compared to household who do not practice urban agriculture. Few studies have investigated intra-urban impact of urban agriculture on U5 malaria unlike rural–urban studies. For example^52^, found a positive association between positive malaria outcome and children living in rural areas of Ghana, as well as^40^ in Mozambique. Rural areas are usually highly vegetated, serving as a suitable habitat for the breeding of mosquitoes. In addition, we do not find an association between the adopted mode of waste disposal method and U5 malaria prevalence. One of the challenges of urbanisation is increasing waste generation as this has consequences on the health of urban residents. Good waste management practices such as regular trash disposal reduce the risk of malaria as there would be less mosquito breeding, clogging and flooding^72^.

Also, the study findings show a non-significant increasing trend in the burden of malaria with each increasing age categories similar to the outcomes observed in the studies of^13,52^. The lower risk of malaria burdens in younger children could be because of the passive immunity acquired from mothers through breastfeeding as observed in the studies of^2,52^. Intuitively, this observed phenomenon might also be due to the fact that older children are less likely to sleep under ITN when there are not enough ITN to serve the younger and older children among poor households.

Lower risk of malaria exists among U5 children in vector-proof houses such as window protection (OR = 0.51; 95% CI = 0.34–0.77). Similar findings are reported by^28,73^. Houses in good condition i.e. mosquito-proofing houses offer significant advantage of equitably protecting all members of particular households even those that are not sleeping under a bed net^73^. Window screening prevent mosquitoes from entering the houses or places of abode. According to our study, households with a piped source of water have reduced odds of U5 malaria (OR = 0.48; 95% CI = 0.31–0.77). In this study, since wealth index was not considered access to piped water is used as a surrogate for wealth index, which explains the reduced odds for households with a piped source of water and window protection. Poor households are likelier to live in substandard houses with avenues for malaria vectors to find their way into the building. These findings are in agreement with the studies of^17,69,74–76^ where highest wealth status households or better off households are noted to afford malaria preventive measures. Some of these measures include appropriate housing facilities with screens that block or hinder vectors resulting in reduced vector-human contact, insecticide-treated bed nets to reduce malaria transmission, quick diagnosis and acquiring of drugs in case of infection without depending on public facilities. Moreover, malaria in Africa have been described as a disease of rural population and communities which are homes of the poorest of the poor^77^, as further illustrated by income level in Table S1 (supplementary file). The higher the income level, the lower the odds of U5 malaria.

### Study limitations and future research

There are some limitations in this study that should be considered when interpreting the study findings. The epidemiologic variable—presence or absence of malaria—retrospectively determined by verbal report might lead to recall bias. Furthermore, not all research variables that influences the transmission of malaria are considered in this study. Therefore, robust health routine survey data with associated environmental factors and SES void of bias should be considered in future study. Nevertheless, this research primarily considered social factors and cross-checked questions on definition of malaria to limit bias was maintained.

It is pertinent to note that the study’s sample size is relatively small with potential to introduce some biases in the study results such as the low proportion of malaria-positive cases. This might have impacted the low Cohens kappa measure i.e. measurement of agreement of the two categorical variable outcomes (positive and negative malaria outcome). However, the obtained results from statistical power analysis test and cross-validation model accuracy have led to improvement of the study validity. Therefore, an extensive future study with more samples should be strongly considered. Lastly, since this is a cross-sectional study, the impact of seasonality on malaria prevalence should be considered while interpreting the results since the burden of malaria varies seasonally.

## Conclusions

This study demonstrated steps toward understanding the spatial structure of U5 malaria through the application of Model-based Geostatistical modelling to a very-fine scale mapping in places of low resource settings such as Akure, Nigeria. The map provides place-based evidence on the spatial variation of U5 malaria in Akure and serves as a guide to locations that require crucial and intensified interventions for the reduction of malaria burden.

The study shows spatially predicted variability of U5 malaria risk in Akure, with high prevalence within the centre of the city, transition zone, and newly developed places/suburbs which are characterised with low urban planning development control. The study further shows low prevalence of U5 malaria burden in the affluent communities such as Alagbaka, Oba-ile etc. According to our findings, the usage of ITNs, window protection, and a piped-water source reduces the risk of U5 malaria. Therefore, interventions addressing these risk factors are germane while also ensuring continuous monitoring of malaria prevalence and intervention assessment should be considered. This is however predicated on the availability of malaria covariates data especially at local level. Hence, barriers on data availability should be addressed. The health challenges of the twenty-first century are complex and requires multiple discipline and approaches to tackle these challenges. Therefore, urban planning control and development in the city core and outlying districts should be intensified.

Geographical or spatial targeting of public health control efforts in U5 malaria hotspots developed in accordance to the exceedance probability model will aid the elimination of malaria in Akure, Nigeria. The evidence-based policy formulation and implementation directed towards places of high malaria risk and transmission can lead to malaria elimination and achieving set target according to the Nigeria’s NMSP. In addition, this can also contribute towards Nigeria’s achievement of Sustainable Development Goals 3 and 11 which are to: (1) Ensure health lives and promote well-being for all at all ages and (2) Making cities and human settlement inclusive, safe resilient and sustainable.

### Supplementary Information

Supplementary Information.

## Data Availability

Data can be made available from the corresponding author upon reasonable request. However, the R scripts for the exploratory analysis, cross-validation, parameter estimation and spatial prediction are freely available at: https://github.com/Taye20/MBG/tree/main.

## Abbreviations

AIC: Akaike information criterion
EP: Exceedance probability
ITN: Insecticide-treated nets
MBG: Model-based Geostatistics
MCML: Monte Carlo maximum likelihood
MIQ: Malaria Indicator Questionnaire
MIS: Malaria Indicator Survey
NMSP: National Malaria Strategic Plan
OR: Odds ratio
SES: Socio-economic status
SDH: Social determinants of health
SGLMMs: Spatial generalised linear mixed models
SSA: Sub- Saharan Africa
U5: Children under five years
VIF: Variance Inflation Factor
WHO: World Health Organization
