# Supplementary material for: Identifying childhood malaria hotspots and risk factors in a Nigerian city using geostatistical modelling approach
Source: Sci Rep. 2024 Mar 5;14:5445. doi: 10.1038/s41598-024-55003-x (PMC10914794; doi:10.1038/s41598-024-55003-x)
Supplement: Supplementary file 1 — Supplementary Information. [file 41598_2024_55003_MOESM1_ESM.docx]

Supplementary Table S1: Description of U5 malaria with considered covariates.

| Factors |  | Negative  (N = 440) | Positive  (N = 128) | OR  (95% CI) | p value |
| --- | --- | --- | --- | --- | --- |
| Drainage Covered |  |  |  |  | 0.023 |
| No |  | 362 (75.7%) | 116 (24.3%) | 1.00 |  |
| Yes |  | 78 (86.7%) | 12 (13.3%) | 0.48 (0.25 – 0.91) |  |
| Toilet Facility |  |  |  |  | 0.229 |
| Flush toilet |  | 361 (78.1%) | 101 (21.9%) | 1.00 |  |
| Bucket toilet |  | 11 (84.6%) | 2 (15.4%) | 0.64 (0.14 – 2.97) |  |
| No facility |  | 1 (33.3%) | 2 (66.7%) | 7 (0.64 – 79.64) |  |
| Pit latrine |  | 67 (74.4%) | 23 (25.6%) | 1.23 (0.73 – 2.07) |  |
| Health Infrastructure |  |  |  |  | 0.372 |
| No |  | 151 (77%) | 45 (23%) | 1.00 |  |
| Not Sure |  | 37 (86%) | 6 (14%) | 0.54 (0.22 – 1.37) |  |
| Yes |  | 252 (76.6%) | 77 (23.4%) | 1.03 (0.67 – 1.56) |  |
| HouseTenure |  |  |  |  | 0.074 |
| Individual ownership |  | 214 (78.1%) | 60 (21.9%) | 1.00 |  |
| Inherited |  | 23 (76.6´7%) | 7 (23.3%) | 1.09 (0.44 – 2.65) |  |
| Others |  | 0 (0%) | 2 (100%) | 17.72 (0.84 – 374.24) |  |
| Rented |  | 203 (77.5%) | 59 (22.5%) | 1.04 (0.69 – 1.56) |  |
| Marital Status |  |  |  |  | 0.489 |
| Divorced |  | 10 (83.3%) | 2 (16.7%) | 1.00 |  |
| Married |  | 387 (76.5%) | 119 (23.5%) | 1.54 (0.33 – 7.11) |  |
| Separated |  | 19 (82.6%) | 4 (17.4%) | 1.05 (0.16 – 6.77) |  |
| Single |  | 18 (85.7%) | 3 (14.3%) | 0.83 (0.12 – 5.85) |  |
| Widowed |  | 6 (100%) | 0 (0%) | 0.32 (0.01 – 7.84) |  |
| Father’s Ethnicity |  |  |  |  | 0.292 |
| Hausa |  | 4 (50%) | 4 (50%) | 1.00 |  |
| Igbo |  | 31 (79.5%) | 8 (20.5%) | 0.26 (0.05 – 1.27) |  |
| Others |  | 8 (72.7%) | 3 (27.3%) | 0.38 (0.05 – 2.55) |  |
| Yoruba |  | 397 (77.8%) | 113 (22.2%) | 0.28 (0.07 – 1.16) |  |
| Mother’s Ethnicity |  |  |  |  | 0.123 |
| Hausa |  | 4 (44.4%) | 5 (55.6%) | 1.00 |  |
| Igbo |  | 46 (78%) | 13 (22%) | 0.23 (0.05 – 0.97) |  |
| Others |  | 9 (675%) | 3 (25%) | 0.27 (0.04 – 1.70) |  |
| Yoruba |  | 381 (78.1%) | 107 (21.9%) | 0.22 (0.05 – 0.85) |  |
| Father’s Education Level |  |  |  |  | 0.297 |
| Apprentice |  | 84 (80.8%) | 20 (19.2%) | 1.00 |  |
| No education |  | 5 (62.5%) | 3 (37.5%) | 2.52 (0.56 – 11.43) |  |
| Primary |  | 14 (70%) | 6 (30%) | 1.80 (0.62 – 5.27) |  |
| Secondary |  | 88 (72.1%) | 34 (27.9%) | 1.62 (0.87 – 3.04) |  |
| Tertiary |  | 249 (79.3%) | 65 (20.7%) | 1.10 (0.63 – 1.92) |  |
| Mother’s Education Level |  |  |  |  | 0.158 |
| Apprentice |  | 95 (77.2%) | 28 (22.8%) | 1.00 |  |
| No education |  | 16 (66.7%) | 8 (33.3%) | 1.70 (0.66 – 4.38) |  |
| Primary |  | 29 (65.9%) | 15 (34.1%) | 1.75 (0.83 – 3.72) |  |
| Secondary |  | 171 (78.1%) | 48 (21.9%) | 0.95 (0.56 – 1.62) |  |
| Tertiary |  | 129 (81.6%) | 29 (18.4%) | 0.76 (0.43 – 1.37) |  |
| Father’s Employment Status |  |  |  |  | 0.053 |
| Formal sector |  | 180 (81.8%) | 40 (18.2%) | 1.00 |  |
| Informal sector |  | 245 (74%) | 86 (26%) | 1.58 (1.04 – 2.41) |  |
| Others |  | 10 (100%) | 0 (0%) | 0.21 (0.01 – 3.70) |  |
| Unemployed |  | 5 (71.4%) | 2 (28.6%) | 1.80 (0.34 – 9.61) |  |
| Mother’s Employment Status |  |  |  |  | 0.051 |
| Formal sector |  | 107 (85.6%) | 18 (14.4%) | 1.00 |  |
| Informal sector |  | 305 (74.6%) | 104 (25.4%) | 2.03 (1.17 – 3.50) |  |
| Others |  | 3 (100%) | 0 (0%) | 0.83 (0.04 – 16.74) |  |
| Unemployed |  | 25 (80.6%) | 6 (19.4%) | 1.43 (0.51 – 3.96) |  |
| Income Level |  |  |  |  | 0.103 |
| < ₦20,000 |  | 33 (70.2%) | 14 (29.8%) | 1.00 |  |
| ₦20,000 - ₦50,000 |  | 162 (76.1%) | 51 (23.9%) | 0.74 (0.37 – 1.50) |  |
| ₦50,000 - ₦100,000 |  | 100 (75.2%) | 33 (24.8%) | 0.78 (0.37 – 1.62) |  |
| ₦100,000 - ₦150,000 |  | 70 (77.8%) | 20 (22.2%) | 0.67 (0.30 – 1.50) |  |
| > ₦150,000 |  | 75 (88.2%) | 10 (11.8%) | 0.31 (0.12 – 0.78) |  |
| Floor covering material |  |  |  |  |  |
| Finished floor |  | 398 (78.2%) | 111 (21.8%) | 1.00 |  |
| Natural floor |  | 22 (81.5%) | 5 (18.5%) | 0.81 (0.30 – 2.20) | 0.105 |
| Rudimentary floor |  | 20 (62.5%) | 12 (37.5%) | 2.15 (1.02 – 4.54) |  |
| Roof covering material |  |  |  |  |  |
| Finished Roof |  | 388 (70%) | 103 (21%) | 1.00 |  |
| Natural Roof |  | 2 (50%) | 2 (50%) | 3.77 (0.52 – 27.07) | 0.103 |
| Others |  | 1 (50%) | 1 (50%) | 3.77 (0.23 – 60.74) |  |
| Rudimentary Roof |  | 49 (69%) | 22 (31%) | 1.69 (0.98 – 2.93) |  |
|  |  |  |  |  |  |


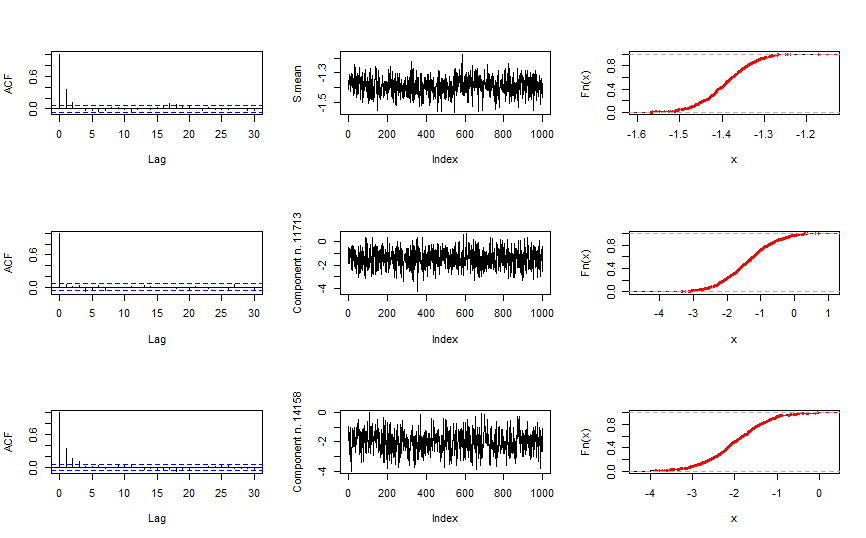


Supplementary Figure S1: Autocorrelation plot of a thinned sequence of 10000 MCMC samples (left panels), trace plot of the same sequence (central panels) and empirical commulative distribution plots for the first 5000 and second 5000 samples (right panels), for the spatial average of predicted logit-transformed prevalence (first row) and for the predicted logit-transformed prevalence at two randomly selected locations (second and third rows). The figure was created with R version 3.6.3, https://cran.rstudio.com/
